# *Abcd1* deficiency accelerates cuprizone-induced oligodendrocyte loss and axonopathy in a demyelinating mouse model of X-linked adrenoleukodystrophy

**DOI:** 10.1186/s40478-023-01595-w

**Published:** 2023-06-18

**Authors:** Ksenija Martinović, Jan Bauer, Markus Kunze, Johannes Berger, Sonja Forss-Petter

**Affiliations:** 1 Department of Pathobiology of the Nervous System, Center for Brain Research, Medical University of Vienna, Spitalgasse 4, 1090 Vienna, Austria; 2 Department of Neuroimmunology, Center for Brain Research, Medical University of Vienna, Spitalgasse 4, 1090 Vienna, Austria

**Keywords:** *Abcd1* KO, Axonopathy, Cuprizone, Demyelination, Microglia, Oligodendrocytes, Peroxisome, X-ALD

## Abstract

X-linked adrenoleukodystrophy (X-ALD), the most frequent, inherited peroxisomal disease, is caused by mutations in the *ABCD1* gene encoding a peroxisomal lipid transporter importing very long-chain fatty acids (VLCFAs) from the cytosol into peroxisomes for degradation via β-oxidation. ABCD1 deficiency results in accumulation of VLCFAs in tissues and body fluids of X-ALD patients with a wide range of phenotypic manifestations. The most severe variant, cerebral X-ALD (CALD) is characterized by progressive inflammation, loss of the myelin-producing oligodendrocytes and demyelination of the cerebral white matter. Whether the oligodendrocyte loss and demyelination in CALD are caused by a primary cell autonomous defect or injury to oligodendrocytes or by a secondary effect of the inflammatory reaction remains unresolved. To address the role of X-ALD oligodendrocytes in demyelinating pathophysiology, we combined the *Abcd1* deficient X-ALD mouse model, in which VLCFAs accumulate without spontaneous demyelination, with the cuprizone model of toxic demyelination. In mice, the copper chelator cuprizone induces reproducible demyelination in the corpus callosum, followed by remyelination upon cuprizone removal. By immunohistochemical analyses of oligodendrocytes, myelin, axonal damage and microglia activation during de-and remyelination, we found that the mature oligodendrocytes of *Abcd1* KO mice are more susceptible to cuprizone-induced cell death compared to WT mice in the early demyelinating phase. Furthermore, this effect was mirrored by a greater extent of acute axonal damage during demyelination in the KO mice. *Abcd1* deficiency did not affect the function of microglia in either phase of the treatment. Also, the proliferation and differentiation of oligodendrocyte precursor cells and remyelination progressed at similar rates in both genotypes. Taken together, our findings point to an effect of *Abcd1* deficiency on mature oligodendrocytes and the oligodendrocyte-axon unit, leading to increased vulnerability in the context of a demyelinating insult.

**Supplementary Information:**

The online version contains supplementary material available at 10.1186/s40478-023-01595-w.

## Introduction

X-linked adrenoleukodystrophy (X-ALD, OMIM #300100) is the most common hereditary peroxisomal disease with an estimated incidence of 1 in 14,700 individuals, including the hemizygous males and heterozygous females [1]. It is caused by mutations in the *ABCD1* gene encoding the peroxisomal lipid transporter ABCD1 (ALD protein) [2, 3]. The lack of ABCD1 function impairs the transport of CoA-activated very long-chain fatty acids (VLCFAs, ≥ C22) into the peroxisomes for degradation via β-oxidation [4], resulting in the accumulation of VLCFAs in tissues and body fluids of X-ALD patients, most notably in severely affected tissues like the brain white matter and the adrenal cortex [5].

X-ALD exhibits a wide range of phenotypic variability with no genotype-to-phenotype correlation [6, 7]. Adrenomyeloneuropathy (AMN), the default, adult onset neurological manifestation affects males and heterozygous females and is characterized by axonopathy of the ascending and descending spinal cord tracts and dysmyelination in the absence of overt cerebral inflammatory demyelination [8]. The most severe X-ALD variant, cerebral ALD (CALD), is characterized by a rapidly progressive, confluent inflammatory demyelination in the brain usually starting in the corpus callosum and spreading bilaterally through both hemispheres [9]. CALD presents most frequently in childhood, but may also affect adolescent or adult males, before or after the onset of AMN. On a histopathological level, CALD lesions involve peripheral immune cell infiltration, microglia and astrocyte activation as well as oligodendrocyte, myelin and axonal loss [10].

The role of VLCFA accumulation in the pathogenesis of X-ALD remains largely unknown. No direct link has been observed between the plasma VLCFA levels in patients and their phenotypes [11]. AMN patients do not exhibit inflammatory demyelination despite having high VLCFA levels, suggesting that additional factors might play a role in the conversion to the inflammatory demyelinating phenotype. The success of hematopoietic stem cell transplantation in treating early stage CALD patients points to the important role of monocytes/macrophages in X-ALD pathology [12, 13]. In line with these findings, X-ALD monocytes are particularly affected by the accumulation of VLCFAs compared to other immune cell types [14]. At baseline, X-ALD monocytes are pro-inflammatory skewed and activated macrophages are less prone to switch to an anti-inflammatory phenotype, further perpetuating the inflammatory response [15].

Whether the CALD-associated oligodendrocyte loss and demyelination stem from a primary intrinsic effect of *ABCD1* deficiency or are the result of a bystander effect of the vehement inflammatory reaction remains highly contested. In human *post-mortem* brain tissue, Eichler and colleagues described a perilesional area characterized by the absence of microglia along with no apparent changes in the myelination status [16]. This area was found immediately beyond the actively demyelinating lesion area characterized by lipid-laden macrophages, suggesting that, contrary to the situation in multiple sclerosis, the inflammatory reaction in CALD trails behind rather than drives the demyelination. Using novel microglia markers for lesion area delineation in CALD *post-mortem* brain tissue, Bergner and colleagues also described microglia loss within the prelesional areas along with acute axonal damage and only minor phenotypic myelin and oligodendrocyte alterations [17]. A follow-up study, comparing lesion architecture in CALD and multiple sclerosis, recapitulated these findings and emphasized the acute axonal damage, axonal loss and relatively minor alterations in oligodendrocytes in prelesional areas devoid of microglia in CALD, suggesting a primary neurodegenerative effect involving the entire axon-myelin unit [18].

To investigate the roles of oligodendrocytes and microglia in the demyelinating pathogenesis of X-ALD, we used the *Abcd1-*deficient mouse model [19]. Mice harboring inactivating mutations in the *Abcd1* gene exhibit VLCFA accumulation similar to human patients and develop a late-onset axonopathy in the spinal cord in the absence of cerebral demyelination, thus approximating some aspects of the AMN phenotype [19–22]. In order to circumvent the lack of spontaneous cerebral demyelination, we treated *Abcd1-*deficient mice with the neurotoxin cuprizone. Administration of cuprizone through diet induces oligodendrocyte death and demyelination in the brain, with the corpus callosum and the superior cerebellar peduncles being the most reproducibly and severely affected areas [23, 24]. Oligodendrocyte and myelin loss are accompanied by microglia activation observable as early as one week after the start of treatment and increasing until cuprizone is removed from the diet [25, 26]. Furthermore, cuprizone withdrawal from the diet induces an almost immediate remyelination of previously demyelinated areas, thus allowing the possibility to study the processes governing regeneration [27]. The cuprizone model has been particularly useful to study the main aspects of pathological responses and mechanisms related to innate immunity such as phagocyte-driven de- and remyelination as well as axonal degeneration in addition to the intrinsic vulnerability of oligodendrocytes and the axon-myelin unit in corpus callosum.

In this, to our knowledge, first report of the cuprizone paradigm in an X-ALD mouse model, we found that *Abcd1*^*−/y*^ mice largely follow the typical temporal and spatial patterns of de- and remyelination, although with a moderately higher susceptibility to cuprizone treatment compared to wild-type (WT) mice. In particular, *Abcd1*^*−/y*^ mice exhibited a more rapid loss of mature oligodendrocytes in the initial stages of demyelination concomitant with a greater extent of acute axonal damage, while the innate immune and myelin responses remained similar between the genotypes. Both *Abcd1*^*−/y*^ and *Abcd1*^+*/y*^ mice exhibit similar regenerative responses, including proliferation of oligodendrocyte precursor cells and generation of new mature oligodendrocytes and myelin sheaths as well as termination of microglia activation and axonal damage.

## Materials and methods

### Mice

The study involved *Abcd1*-deficient (B6.129-Abcd1^tm1Kan/J^) X-ALD mice [19], backcrossed on the C57BL/6J background for more than 20 generations, and wild-type C57BL/6J mice. Experimental cohorts, consisting of adult male *Abcd1*^+*/y*^ (WT) and *Abcd1*^*−/y*^ (KO) litttermates, were generated by breeding *Abcd1*^+/-^ females and C57BL/6J males. Primers and conditions for genotyping have been described previously [28]. Mice were group housed at the local animal facility of the Medical University of Vienna in an environmentally-controlled room on a 12:12 h light–dark cycle and with ad libitum access to food and water. Before and after receiving the cuprizone diet, the mice were maintained on standard mouse breeding chow (M-Z, Ssniff®, Soest, Germany).

### Cuprizone treatment

**Fig. 1:**
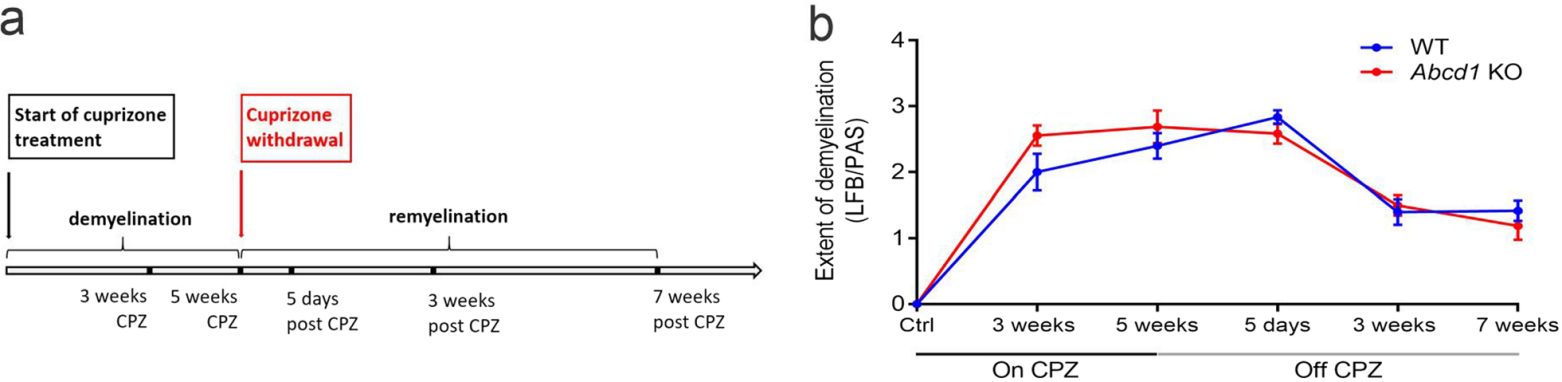
Timeline of the cuprizone administration and myelination state in *Abcd1* KO and WT mice. (**a**) Schematic depicting the cuprizone-feeding paradigm used in the study, along with the corresponding time points for analysis. (**b**) Evaluation of the extent of demyelination and remyelination in the corpus callosum based on LFB/PAS myelin staining (subjective score: 0, complete myelination; 3, complete demyelination). The graph shows group means ± SEM (controls: *n* = 5 WT, 4 *Abcd1* KO; treatment time points: *n* = 6–10 WT, 6–9 *Abcd1* KO mice). The individual values are displayed in Additional file 2:  Fig. S2b

Ten-weeks (± 2 days) old, male *Abcd1* WT and KO mice were placed on a diet containing 0.3% (w/w) cuprizone (CPZ, bis-cyclohexanone-oxaldihydrazone, Sigma-Aldrich, Germany) pressed into the M-Z chow pellets (custom made by Ssniff)] for a maximum of 5 weeks to induce demyelination in the corpus callosum [29–31]. After cuprizone removal, the mice were allowed to recover on standard chow diet for a maximum of 7 weeks. Food consumption/cage and body weight (Additional file 2: Fig. S2) were monitored regularly. None of animals showed sickness behavior or died during the study. A single mouse was discontinued prematurely due to weight loss criteria. Mice were terminated for immunohistochemical analyses at several time points (*n* = 6–10 per time point and genotype) during the demyelination (3 and 5 weeks CPZ) and remyelination (5 days, 3 and 7 weeks off CPZ) phase (Fig. 1). Three animals (1 WT at 3 weeks CPZ; 1 WT and 1 KO at 5 weeks CPZ) were excluded from the study due to an undetectable treatment response, possibly due to technical failure during sample handling. Exclusion criteria required a lack of all major pathological hallmarks of the model: microglia activation, myelin and oligodendrocyte loss, and acute axonal damage. Untreated control animals were sacrificed at 13 weeks of age, corresponding to the first (3-week) time point for analysis of the cuprizone-fed mice. In addition, immunohistochemical analyses of corpus callosum were performed in untreated 20–22 months old *Abcd1* WT and KO mice.

The mouse study was performed in compliance with the 3Rs of animal welfare (replacement, reduction, refinement) and animal policies for humane care and handling according to the national (Austrian) regulations (BGBl. II Nr. 522/2012) and the European Union directive 2010/63/EU. Ethical approval for the study and procedures for the mice was obtained from the Institutional Animal Care and Use Committee of the Medical University of Vienna and the Austrian Federal Ministry of Science, Research and Economy (BMBWF-66.009/0174-V/3b/2019).

### Histology and immunohistochemistry (IHC)

Mice were transcardially perfused with PBS, followed by 4% paraformaldehyde (PFA) in phosphate-buffered saline (PBS, pH 7.4) under deep ketamine/xylazine-induced anesthesia. The brains were removed, post-fixed in 4% PFA overnight, cut into coronal slices (2–4 mm thickness) using a brain mold and paraffin-embedded. For histology and IHC, coronal paraffin sections (2–4 µm) were collected at the level of the caudal corpus callosum above the hippocampus. For standard neuropathological evaluation, tissue sections were stained with hematoxylin and eosin (H&E) and Luxol fast blue/Periodic acid Schiff (LFB/PAS) for detection of total cell numbers and myelin sheaths, respectively. For antibody application, paraffin sections were deparaffinized and rehydrated, followed by blocking of endogenous peroxidase activity with 0.2% H_2_O_2_ in methanol for 30 min. Antigen retrieval was done in a household food steamer device (MultiGourmet FS 20, Braun, Germany) by 60 min incubation in EDTA (0.05 M) in TRIS buffer (0.01 M, pH 8.5) or citrate buffer (pH 6), followed by a 15-min blocking step with 10% FCS/DAKO and primary antibody incubation at 4 °C overnight. Secondary antibodies were incubated for 1 h at room temperature, followed by a 1-h incubation with avidin-peroxidase at room temperature and detection using 3,3’-diaminobenzidine (DAB) and H_2_O_2_ in PBS.

### Immunofluorescence and confocal microscopy

The tissue sections were deparaffinized and rehydrated, followed by a 1-h EDTA pretreatment (pH 8.5) in the steamer. Blocking was done in Dako diluent for 20 min followed by the OLIG2 primary antibody application at 4 °C overnight. Secondary, biotinylated donkey anti-rabbit antibody was incubated for 1 h at room temperature, followed by a 1-h incubation with avidin-peroxidase at room temperature. Signal enhancement was done using biotinylated tyramide (CSA) for 20 min at room temperature. Sections were rinsed in PBS and steamed for 30 min in (EDTA pH 8.5), followed by primary antibody application (sheep-anti CAII and rabbit-anti GFAP) at 4 °C overnight. Secondary antibodies were subsequently incubated for 1 h at room temperature. Stained sections were scanned using a Vectra Polaris slide scanner (Akoya Biosciences).

### Antibodies

The following primary antibodies were used for the immunohistochemical analyses: amyloid precursor protein (APP, mouse, 1:1000, Millipore); proteolipid protein (PLP, rabbit, 1:250, abcam), carbonic anhydrase II (CAII, sheep, 1:1000, BindingSite), oligodendrocyte transcription factor 2 (OLIG2, rabbit, 1:1000, Millipore), ionized calcium-binding adaptor molecule 1 (IBA1, rabbit, 1:3000, Wako), MAC3 (rat, 1:100, BD Pharmingen™) and glial fibrillary acidic protein (GFAP, rabbit, 1:3000, Dako). The following secondary antibodies were used: biotinylated donkey anti-mouse (Jackson ImmunoResearch Laboratories, 1:500), biotinylated donkey anti-rat (Jackson ImmunoResearch Laboratories, 1:500), biotinylated donkey anti-sheep (Jackson Laboratories, 1:500), biotinylated donkey anti-rabbit (Jackson Laboratories, 1:1000). Peroxidase-conjugated streptavidin: Jackson ImmunoResearch Laboratories, dilution: 1:500. For confocal microscopy, the following primary antibodies were used: glial fibrillary acidic protein (GFAP, rabbit, 1:1000, Dako), OLIG2 (rabbit, 1:7000, Millipore), CAII (sheep, 1:250, BindingSite). The following secondary antibodies were used: biotinylated donkey anti-rabbit, (Jackson ImmunoResearch Laboratories, 1:1000), Streptavidin-Cy2 (Jackson ImmunoResearch Laboratories, 1:100); donkey anti-sheep-Cy3 (Jackson ImmunoResearch Laboratories, 1:100) and donkey anti-rabbit-Cy5 (Jackson ImmunoResearch Laboratories, 1:100).

### IHC quantification

Stained sections were scanned using a Hamamatsu NanoZoomer 2.0 HT scanner (brightfield settings, magnification: 40×) and analyzed using ImageJ and QuPath. Comparative analyses were performed on the medial part of the corpus callosum above the fornix with a fixed surface area of 0.04 mm^2^. The extent of myelin loss in LFB/PAS stainings was evaluated subjectively with genotypes blinded using a 0–3 scoring system (0: no demyelination, 3: full demyelination). Quantification of CAII- and OLIG2-positive oligodendrocyte numbers was performed in QuPath using variable detection thresholds and expressed as positive cells/mm^2^. For IHC of IBA1, MAC3 and PLP, the DAB^+^ staining was extracted in QuPath and further analyzed in ImageJ using macro software with constant thresholds; the results are shown as % immunoreactive area of total area. Quantification of APP stainings was performed in QuPath, and the total numbers of axonal spheroids accumulating APP are expressed as spheroids/mm^2^.

### Statistics

All statistical analyses were carried out using the GraphPad Prism 7 software (GraphPad Software, San Diego, California). The data is displayed as box plots according to Tukey (with dot plot overlays) showing medians and error bars corresponding to 1.5 IQR. Grubbs outlier test was performed on all data sets and the identified outliers were excluded from the statistics, as indicated in the figure legends. One-way ANOVA with Sidak’s multiple comparisons test was performed to compare the genotype responses at different treatment time points. Adjusted *p*-values are shown as follows: *p < 0.05, ****p < 0.0001.

### Gene expression analysis

Mice were euthanized by using CO_2_ inhalation, and the brain was collected. After cooling the tissue in ice-cold PBS, the rostral and caudal parts of the corpus callosum were dissected from coronal brain slices, immediately snap-frozen in liquid N_2_ and stored at −80 °C. RNA was isolated from corpus callosum samples homogenized in Trizol™ Reagent (Invitrogen) and further purified using RNeasy Mini Kit (Qiagen) columns according to manufacturers´ instructions. Concentration and quality of RNA were determined using a NanoDrop 2000c Spectrophotometer (Thermo Scientific). From 500 ng total RNA, cDNA was synthesis using the iScript cDNA synthesis kit (Bio-Rad). Quantitative PCRs were done as single measurements using aliquots corresponding to 25 ng RNA with SsoFast EvaGreen Supermix on a CFX96 Real-Time PCR Detection System (Bio-Rad). Relative changes in gene expression were obtained using the 2^–∆∆Ct^ method (Schmittgen & Livak, 2008). The relative expression levels (Ct values) of each gene were normalized to that of the housekeeping gene *Hprt* and calibrated to one designated control sample (adjustment for between-plate variation). Expression levels of the following genes were measured: *Car2* (carbonic anhydrase 2), *Olig2* (oligodendrocyte transcription factor 2), *Plp1* (proteolipid protein 1), *Aif1* (allograft inflammatory factor 1, alias *Iba1*), *Gfap* (glial fibrillary acidic protein) and *Hprt* (hypoxanthine guanine phosphoribosyltransferase). The PCR primers are listed in Additional file 1:  Table S1.

## Results

### *Abcd1* KO mice show the typical spatial and temporal demyelination and remyelination responses to acute cuprizone intoxication

Previous research suggests that even up to 21-month-old *Abcd1* KO mice do not develop signs of myelin disturbance or immune cell infiltration in the brain [22, 28]. In line with these findings, our immunohistochemical analyses of myelin and oligodendrocyte proteins, microglia/macrophage markers and APP-positive axonal spheroids in the corpus callosum of 21-mon-old *Abcd1* KO versus WT mice revealed no signs of demyelination, axonopathy or elevated microglia/macrophage activation (Additional file 2: Fig. S1).

Given the absence of spontaneous cerebral involvement in both young and old *Abcd1* KO mice, we proceeded to induce cerebral demyelination by applying the cuprizone model to young, 10-weeks-old, WT and *Abcd1* KO mice (Fig. 1a). Because there are no previous reports of the cuprizone model applied to X-ALD mice, we first investigated whether *Abcd1* KO mice fed a cuprizone diet for 5 weeks, followed by standard chow for up to 7 weeks, develop the expected pattern of demyelination and remyelination in the corpus callosum. We opted for the 5-week cuprizone treatment to achieve acute demyelination, thereby avoiding the spontaneous remyelination observed after 6 weeks [32, 33]. Mice were terminated for histochemical analyses after 3 and 5 weeks on 0.3% cuprizone and after 5 days, 3 weeks and 7 weeks of recovery (Additional file 2: Fig. S2a, b). We observed no major side effects from administering cuprizone and, after an expected initial drop, body weights stabilized or increased (Additional file 2: Fig. S2c). Our results from the subjective scoring of the LFB/PAS myelin stain in both *Abcd1* KO and WT mice indicated a demyelinating response congruent with that reported in the literature (Fig. 1b, Additional file 2: Fig. S2a, b). Compared to WT, a larger fraction of the *Abcd1* KO mice appeared to be almost completely demyelinated (scores of 2.5–3) in the medial corpus callosum already at 3 weeks (Additional file 2: Fig. S2b). Furthermore, upon cuprizone withdrawal, both genotypes showed robust remyelination, which was still incomplete 7 weeks after terminating the intoxication.

### *Abcd1* KO oligodendrocytes and axons show increased vulnerability to cuprizone exposure

**Fig. 2:**
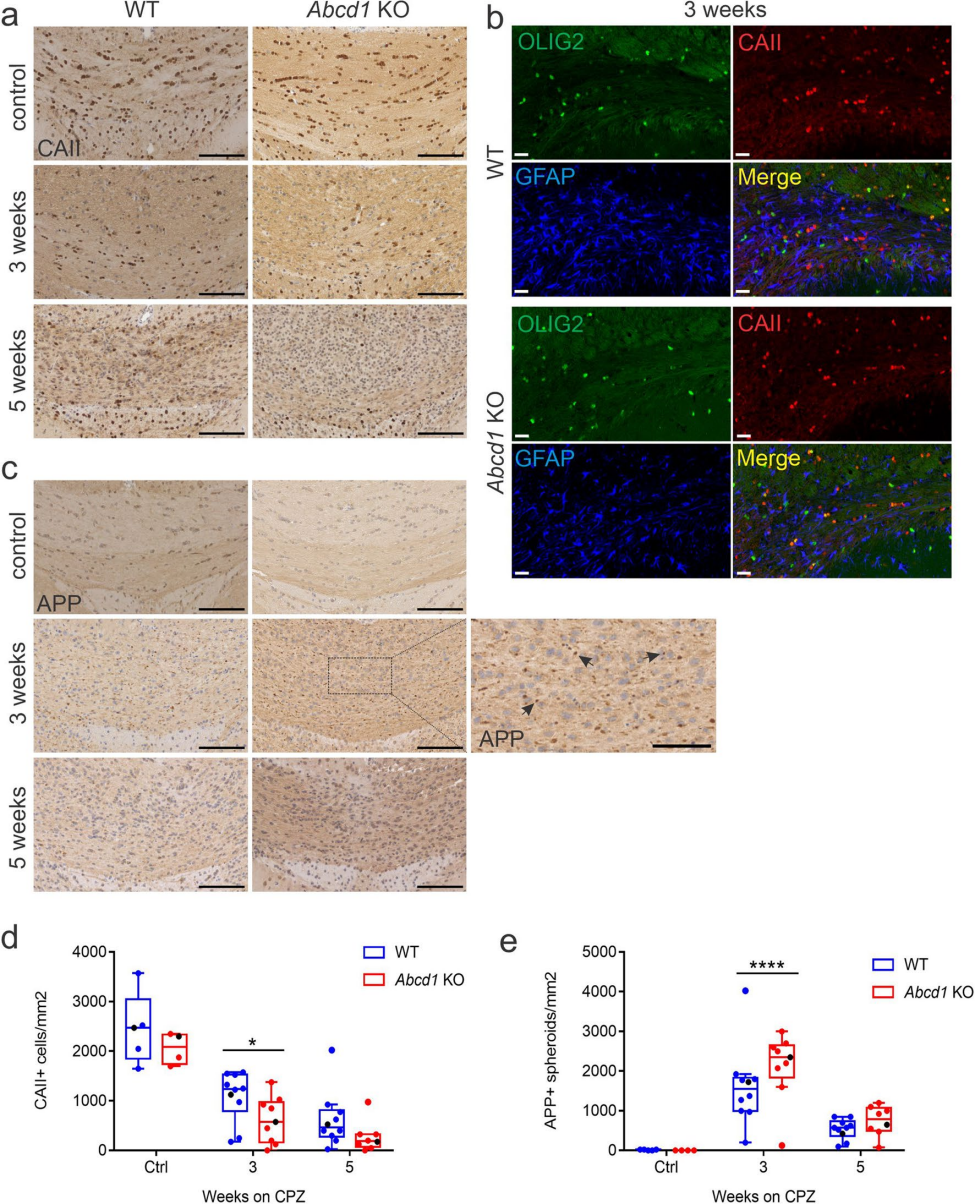
Accelerated oligodendrocyte loss and axonopathy in cuprizone-intoxicated *Abcd1* KO mice. (**a**) Representative images of CAII immunohistochemistry showing mature oligodendrocytes at control, 3 and 5 weeks cuprizone (CPZ) treatment in the medial corpus callosum of WT and *Abcd1* KO mice. Scale bar: 100 µm. (**b**) Confocal triple staining showing mature oligodendrocytes (CAII, red), all cells of the oligodendrocytic lineage (Olig2, green) and astrocytes (GFAP, blue) at 3 weeks CPZ, WT and *Abcd1* KO mice (scale bar: 20 µm). (**c**) Micrographs showing APP^+^ axonal spheroids revealing acute axonal damage as described in (a). Scale bar: 100 µm. Magnified view of the 3 weeks CPZ, *Abcd1* KO; scale bar: 50 µm. (**d**, **e**) Quantifications of the number of CAII^+^ cells and APP^+^ spheroids are shown as box plots according to Tukey with dot plot overlays, medians and 1.5 IQR error bars. The data points represented in the micrographs are color-coded in black. Grubbs outlier test detected two outliers at 3 weeks CPZ for APP and two at 5 weeks CPZ for CAII, shown in the graphs, but excluded from the subsequent statistical analysis. Statistics: One-way ANOVA with Sidak’s multiple comparisons test. Controls: *n* = 5 WT, 4 *Abcd1* KO; 3 weeks CPZ: *n* = 10 WT, 9 *Abcd1* KO; 5 weeks CPZ: *n* = 10 WT, 8 *Abcd1* KO. Adjusted *p*-value: *p = 0.0226; ****p < 0.0001)

We next investigated the response to cuprizone administration in the oligodendrocyte lineage of X-ALD mice in more detail. Oligodendrocyte cell death starts during the first week of cuprizone intoxication, with the extent of cell loss increasing with ongoing treatment [34, 35]. With metabolic and mitochondrial disturbances reported in *Abcd1* deficiency [36] as well as in cuprizone-treated oligodendrocytes [31], we hypothesized that the *Abcd1* defect would render oligodendrocytes more susceptible to the treatment. Detection of mature oligodendrocytes by immunohistochemistry for CAII revealed fewer positive cells in the medial corpus callosum of *Abcd1* KO mice compared to WT littermates after 3 and 5 weeks on cuprizone (Fig. 2a, d). In order to validate the CAII antibody as a selective marker for mature oligodendrocytes, we performed fluorescent triple stainings with CAII, OLIG2 and GFAP. In both WT and *Abcd1* KO mice at 3 weeks of cuprizone treatment, the CAII antibody colocalized with the oligodendrocyte pan-marker OLIG2 but not with GFAP^+^ astrocytes, confirming the specificity (Fig. 2b). Taken together, these data indicate that *Abcd1* deficient oligodendrocytes exhibit a stronger response to cuprizone resulting in a faster drop in mature oligodendrocyte numbers. In addition to the loss of mature oligodendrocytes, cuprizone administration is associated with axonal pathology [27]. In our study, as reflected by IHC detection of APP-positive axonal spheroids, the initial decline in mature oligodendrocyte numbers was associated with a concomitant increase in the extent of acute axonal damage in both genotypes, with the *Abcd1* KO mice being more severely affected already at 3 weeks (Fig. 2 c, e). Taken together, these results suggest that *Abcd1* deficiency renders oligodendrocytes more vulnerable to the cuprizone treatment and, furthermore, that this effect is mirrored by a greater extent of axonal damage.

### The dynamics of myelin loss and microglia activation during cuprizone-induced demyelination are similar in WT and *Abcd1* KO mice

**Fig. 3:**
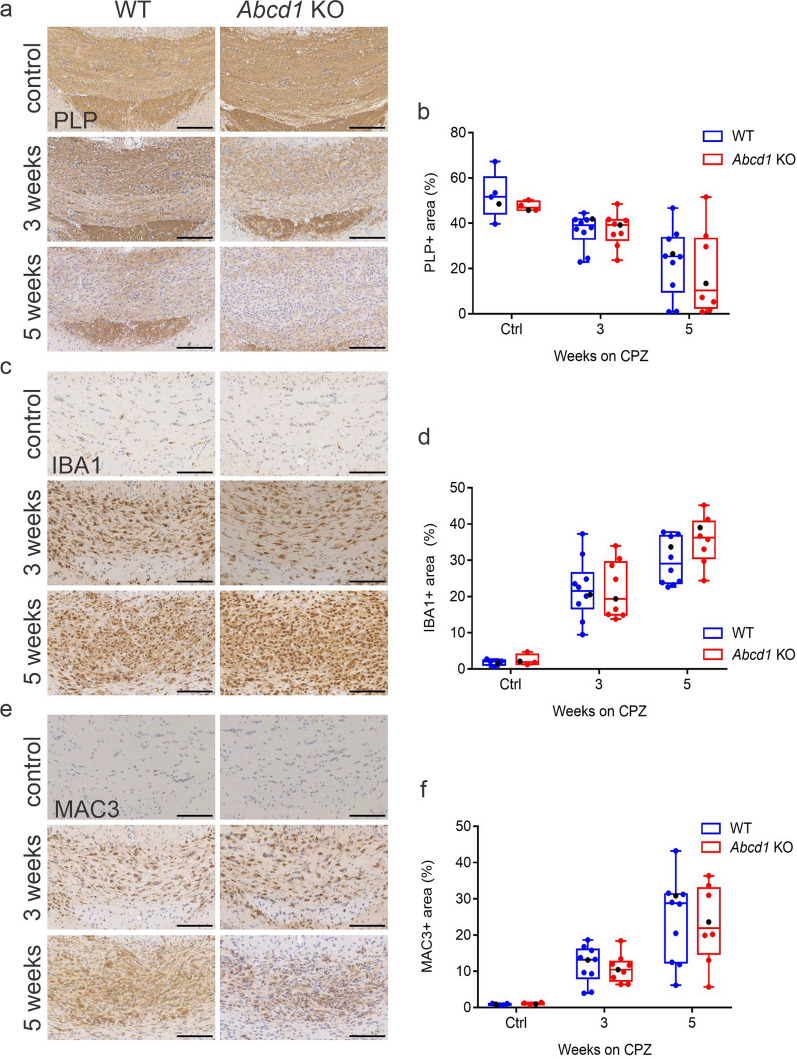
WT and *Abcd1* KO mice show similar dynamics of myelin loss and microglia activation during cuprizone-induced demyelination. (**a**–**f**) Representative images of the medial corpus callosum of WT and *Abcd1* KO mice showing immunohistochemistry for the myelin protein PLP (**a**), total (IBA1^+^) microglia (**c**) and activated (MAC3^+^) microglia (**e**) and quantification at baseline, 3 weeks and 5 weeks of cuprizone treatment. Scale bar: 100 µm. Quantification of the PLP^+^ (**b**), IBA1^+^ (**d**) and MAC3^+^ (**f**) staining is displayed as % of total area analyzed. The data are shown as box plots according to Tukey with dot plot overlays, medians and 1.5 IQR error bars. The data points represented in the micrographs are color-coded in black. Statistical analysis: One-way ANOVA with Sidak’s multiple comparisons test. Controls: *n* = 5 WT, 4 *Abcd1* KO; 3 weeks CPZ: *n* = 10 WT, 9 *Abcd1* KO; 5 weeks CPZ: *n* = 10 WT, 8 *Abcd1* KO

In addition to early oligodendrocyte loss, the cuprizone model is characterized by the progressive loss of myelin sheaths [25]. By IHC for the major myelin protein PLP, we observed the expected increase in myelin loss in both genotypes during the demyelinating phase of the treatment. However, contrary to the loss of oligodendrocyte cell bodies, neither at 3 nor 5 weeks of cuprizone diet could we detect any statistically significant differences in the extent of myelin loss, quantified as PLP-positive surface area, between *Abcd1* KO and WT mice (Fig. 3a, b).

Next, we assessed the extent of microglial activation during the demyelinating phase of cuprizone treatment. We observed the expected dynamics of microglia recruitment and activation in both genotypes, reflected in the progressive increase in surface area occupied by total (IBA1-positive) as well as activated (MAC3-positive) microglia in the medial corpus callosum (Fig. 3c–f). Furthermore, the activation trajectories were similar, and we observed no apparent morphological differences in the microglia between the genotypes.

Taken together, our results indicate that *Abcd1* deficiency does not substantially affect the microglial responses to cuprizone or the myelin dynamics within the demyelinating lesions.

### Cuprizone treatment induces similar astrocytic responses in WT and *Abcd1* KO mice

**Fig. 4:**
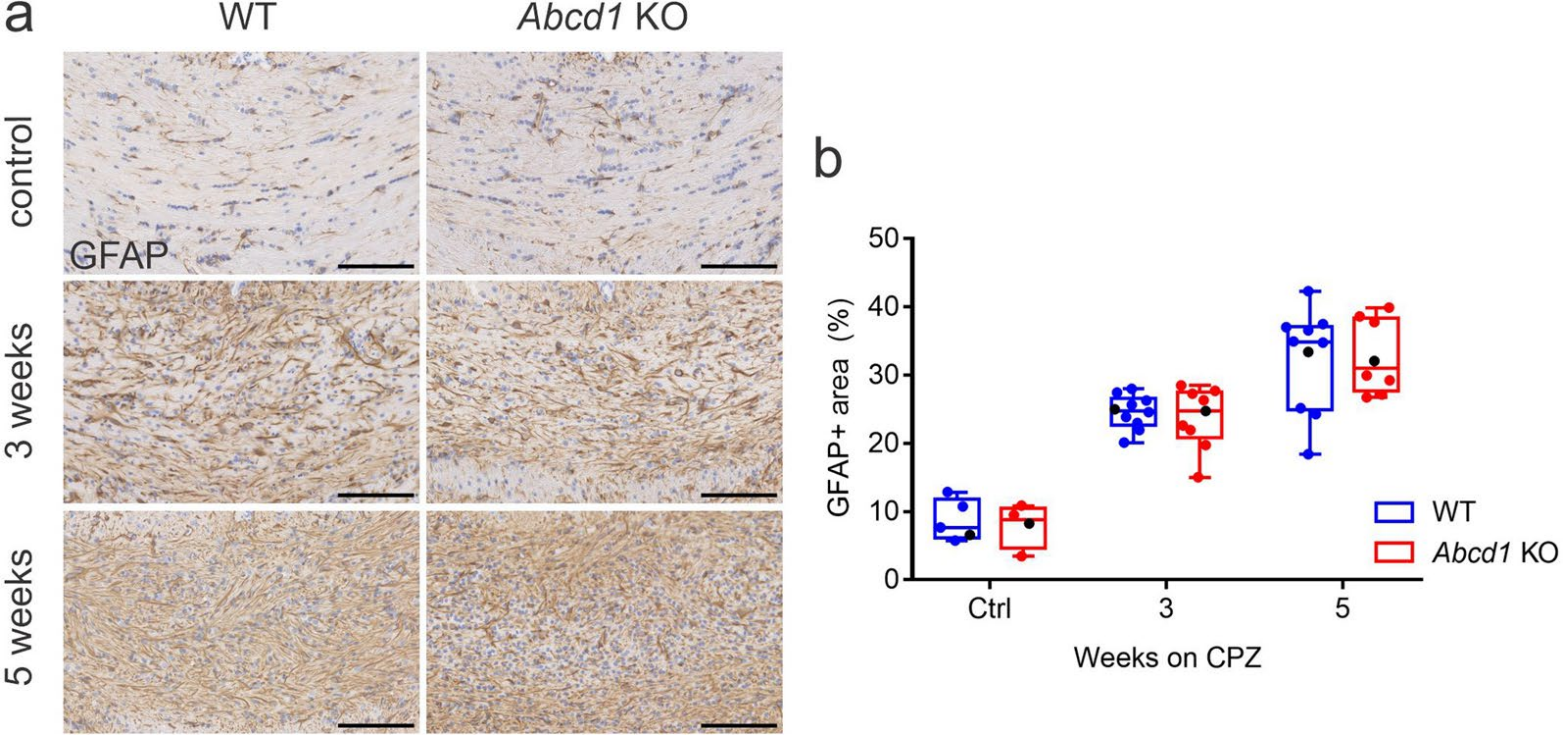
WT and *Abcd1* KO mice show similar extent of astrocyte activation during cuprizone-induced demyelination. (**a**) Representative immunohistochemistry images for GFAP in the medial corpus callosum of WT and *Abcd1* KO mice at baseline, 3 weeks and 5 weeks of cuprizone treatment. Scale bar: 100 µm. (**b**) Quantification of GFAP staining is displayed as % of the total area analyzed. The data are depicted as box plots according to Tukey with dot plot overlays, medians and 1.5 IQR error bars. The data points represented in the micrographs are colod-coded in black. Statistical analysis: One-way ANOVA with Sidak’s multiple comparisons test. Controls: *n* = 5 WT, 4 *Abcd1* KO; 3 weeks CPZ: *n* = 10 WT, 9 *Abcd1* KO; 5 weeks CPZ: *n* = 10 WT, 8 *Abcd1* KO

Cuprizone-induced demyelination is also associated with activation and proliferation of astrocytes [26]. Immunohistochemistry for GFAP, a hallmark cytoskeletal protein which is upregulated in reactive astrocytes, revealed strongly increased staining in the lesion area of corpus callosum in both WT and *Abcd1* KO mice in response to treatment (Fig. 4a, b). Furthermore, the two genotypes exhibited a similar extent of astrocyte activation at both 3 and 5 weeks of cuprizone diet, and no apparent morphological differences were observed between the WT and KO groups. Taken together, our results suggest that *Abcd1* deficiency does not have an impact on astrocytic responses to cuprizone intoxication and the induced demyelination.

### OPC recruitment and remyelination progress at similar rates in *Abcd1* KO and WT mice

**Fig. 5:**
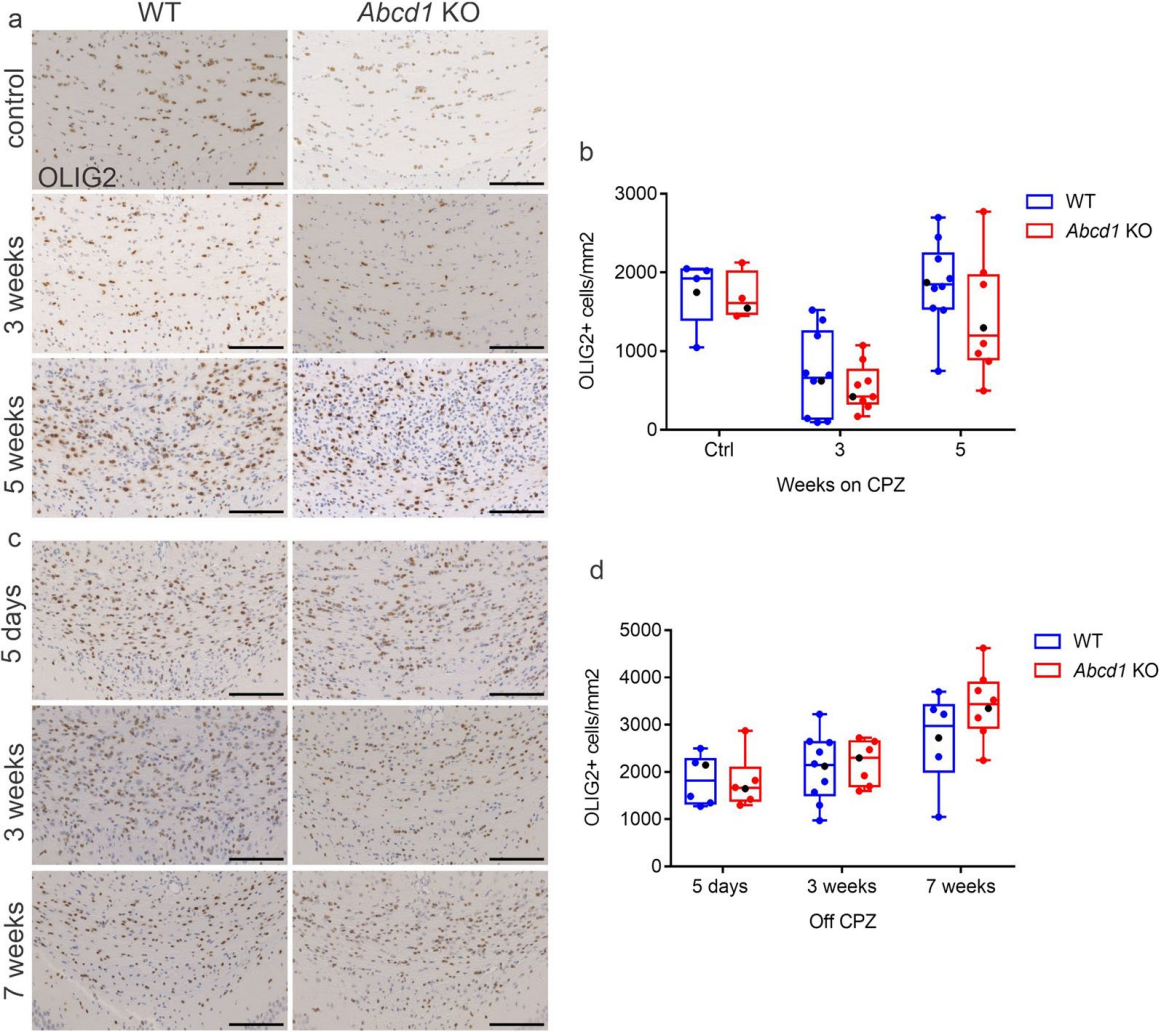
*Abcd1* KO OPCs proliferate normally in response to cuprizone intoxication. (**a**–**d**) Representative images of immunohistochemistry for OLIG2 in the corpus callosum of WT and *Abcd1* KO mice before, during (**a**) and after (**c**) dietary cuprizone administration showing all cells of the oligodendrocyte lineage. Scale bar: 100 µm. Quantifications of OLIG2^+^ cell density (cells/mm^2^) in the demyelination (**b**) and remyelination (**d**) stages are depicted as box plots according to Tukey with dot plot overlays, medians and 1.5 IQR error bars. The data points represented in the micrographs are color-coded in black. Statistical analysis: One-way ANOVA with Sidak’s multiple comparisons test (*n* = 5–10 WT, 4–10 *Abcd1* KO mice)

**Fig. 6:**
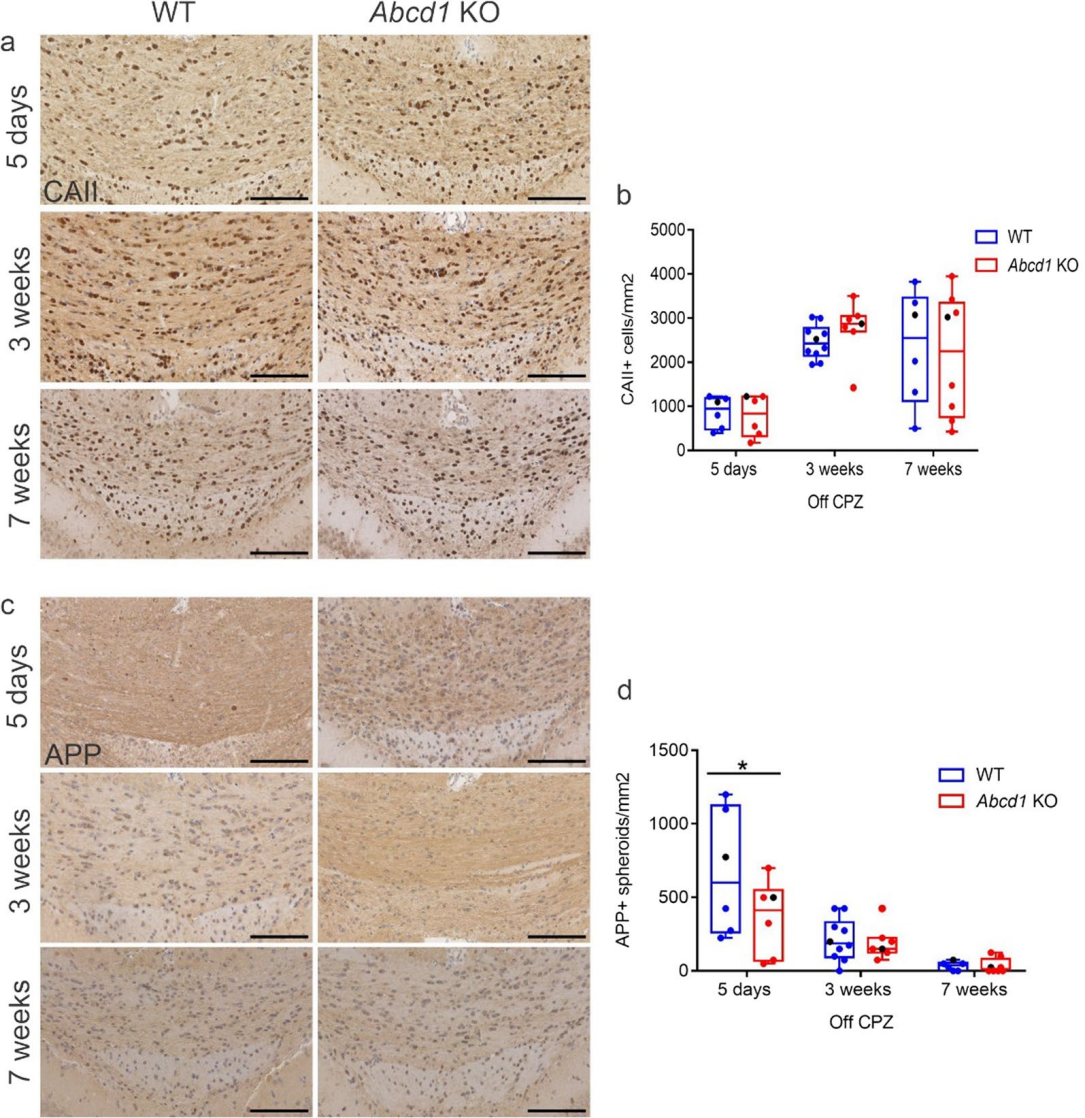
Mature oligodendrocyte numbers increase to a similar extent in WT and *Abcd1* KO mice upon cuprizone withdrawal. (**a**) Representative images of immunohistochemistry for in the corpus callosum of WT and *Abcd1* KO mice during recovery from 5 weeks cuprizone intoxication show mature (CAII^+^) oligodendrocytes (**a**) and of APP^+^ spheroids marking acute axonal damage (**c**). Scale bar: 100 µm. (**b**), (**d**) Quantifications of (**a**) and (**c**), respectively. Statistical analysis: One-way ANOVA with Sidak’s multiple comparisons test (*n* = 6–10 WT; 6–8 *Abcd1* KO mice). For APP 5 days off CPZ, adjusted *p*-value: *p = 0.0353

In the cuprizone model, remyelination is initiated while active demyelination is still ongoing [37]. This process entails a series of steps ranging from oligodendrocyte precursor cell (OPC) recruitment, proliferation and activation to the generation of mature oligodendrocytes and the synthesis of new myelin sheath [38, 39]. Immunohistochemical analysis of OLIG2, a transcription factor that defines the oligodendrocyte lineage and is highly expressed in OPCs, revealed similar densities of OLIG2^+^ cells in WT and *Abcd1* KO mice before and during cuprizone treatment (Fig. 5a, b). Following a marked drop at 3 weeks of cuprizone exposure, reflecting the loss of mature oligodendrocytes, the number of OLIG2-expressing cells increased rapidly. Already 5 days after removal of cuprizone from the diet, the untreated control values were reached in both genotypes and the rate of increase tapered off, with parallel trajectories (Fig. 5c, d). Thus, total oligodendrocyte numbers indicated normal activation and proliferation of OPCs in *Abcd1* deficiency. Immunohistochemistry for CAII in the remyelination phase revealed an increase also in mature oligodendrocyte numbers, indicative of successful oligodendrocyte differentiation and maturation processes (Fig. 6a, b).

Furthermore, the extent of acute axonal damage (APP^+^ spheroids) declined in both genotypes in the post-cuprizone phase and approached untreated controls level after 7 weeks of recovery (Fig. 6c, d). Interestingly, we observed a smaller degree of ongoing axonal damage in *Abcd1* KO compared to wild-type mice 5 days after cuprizone withdrawal. Taken together, our results suggest that *Abcd1* deficiency does not hinder the capacity of OPCs to proliferate and differentiate in response to cuprizone-induced demyelination.

**Fig. 7:**
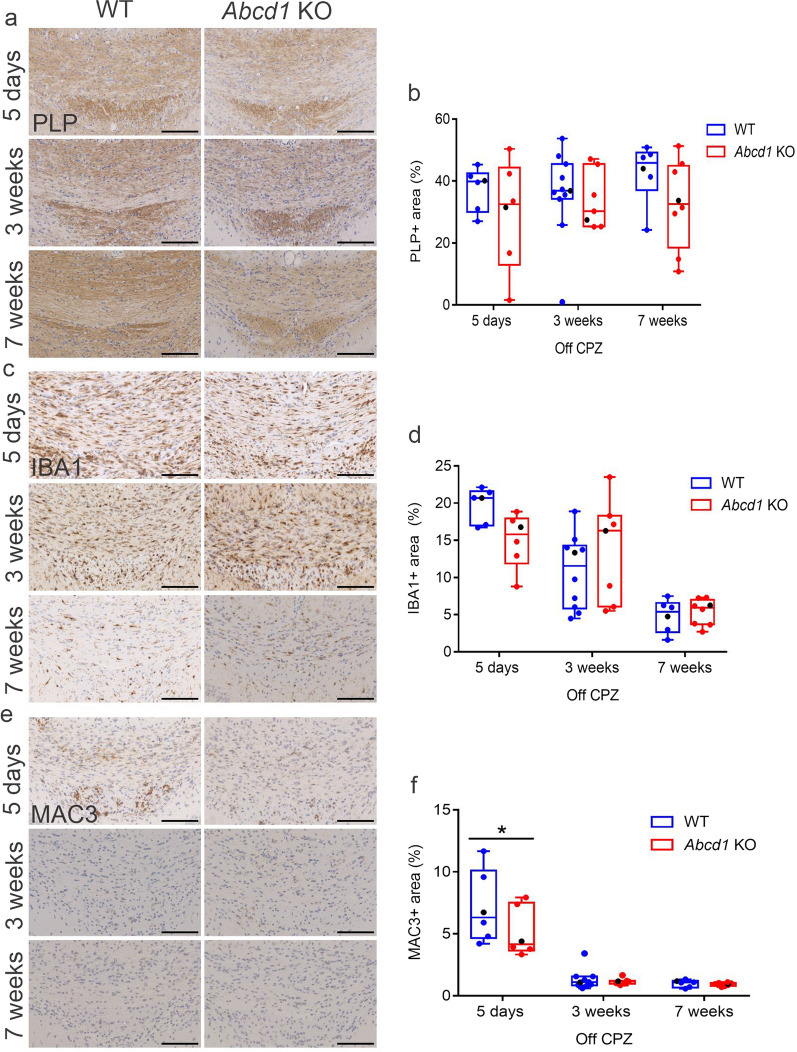
Attenuation of microglial response and myelin renewal are not impaired in *Abcd1* KO mice. Representative images of the corpus callosum of WT and *Abcd1* KO mice after cuprizone withdrawal showing immunohistochemical detection and quantification of (**a**, **b**) PLP^+^ myelin, (**c**, **d**) total (IBA1^+^) microglia and (**e**, **f**) activated (MAC3^+^) microglia at 5 days, 3 weeks and 7 weeks post cuprizone (CPZ). Scale bar: 100 µm. Two outliers detected by the Grubbs outlier test at 3 weeks off CPZ (one each from the PLP WT and MAC3 WT groups) are shown in the graphs but were excluded from the statistics. Statistical analysis: One-way ANOVA with Sidak’s multiple comparisons test (*n* = 6–10 WT; 6–8 *Abcd1* KO mice). For MAC3 5 days off CPZ, adjusted *p*-value: *p = 0.0366

During the recovery phase, *Abcd1* KO mice generated new PLP-positive myelin sheaths to an extent comparable to the WT mice (Fig. 7a, b). However, here the high variability in the extent and/or intensity of the PLP staining within the groups may have precluded the detection of small differences.

In addition to the generation of new myelin sheaths, cuprizone removal from the diet triggers a concomitant attenuation of the microglia response [26]. We observed the typical decrease in the staining of total (IBA1^+^) and activated (MAC3^+^) microglia in both genotypes after cuprizone withdrawal (Fig. 7c–f). Analogous to a smaller extent of ongoing acute axonal damage in the *Abcd1* KO mice 5 days after cuprizone removal, we observed less microglial activation in the KO mice compared to WT at this time point (Fig. 7f). This was mirrored by a tendency towards an overall lower microglia (IBA1^+^)-covered surface area. During the later stages of remyelination, the microglia dynamics were similar in both genotypes. Taken together, our results suggest that *Abcd1* deficiency in mice does not impede the resolution of microglial activation and the generation of new myelin sheaths and that the resolution of the innate immune response may even be initiated sooner than in WT mice.

### The mRNA levels of oligodendrocyte/myelin, microglia and astrocyte genes are similarly affected by cuprizone in *Abcd1* KO and WT mice

Gene expression analysis of the *Car2,* O*lig2, Plp1, Iba1/Aif1* and *Gfap* mRNA levels generally revealed cuprizone-dependent effects reflecting the profiles of the corresponding proteins across the treatment paradigm. However, no consistent differences could be detected between the genotypes, except for *Aif1* expression which appeared to be higher (adjusted *p* = 0.0497) *in* the KO mice during the late (5 weeks CPZ) stage of demyelination (Additional file 2: Fig. S3).

## Discussion

The most prominent histopathological feature of cerebral ALD is the inflammatory demyelination associated with extensive oligodendrocyte cell death [10, 40, 41]. Despite the important role of oligodendrocytes in the pathophysiology of CALD, the effects of *ABCD1* deficiency on their function remain largely unknown. This study addressed the role of oligodendrocytes lacking ABCD1 in a demyelinating context by combining the X-ALD mouse model harboring an *Abcd1* null mutation [19] with the cuprizone model of toxic demyelination [24].

Our characterization of the cuprizone-induced demyelination response showed the expected loss of mature oligodendrocytes and myelin in the corpus callosum of *Abcd1* KO mice. However, we observed a more pronounced decline in mature oligodendrocyte numbers in *Abcd1* KO mice compared to WT mice in the early phase (at 3 weeks) of cuprizone exposure. This was mirrored by a greater extent of acute axonal damage in *Abcd1* KO mice. There could be several reasons for this observation. Firstly, oligodendrocytes and axons are metabolically coupled, whereby oligodendrocytes provide the axons with trophic support in the form of lactate or pyruvate [42–44]. This metabolic coupling appears to be independent of the structural integrity of myelin, as mice lacking the myelin proteins PLP or CNP exhibit axonal degeneration despite normal-appearing myelin structure [45, 46]. In light of these findings, it is conceivable that metabolic disturbances in X-ALD oligodendrocytes could have an adverse effect on the axons by decreasing the amount of trophic support and compromising their transport function. Furthermore, such effects could be exacerbated by the application of cuprizone imposing additional metabolic burden on the oligodendrocytes. Alternatively, cuprizone itself could be causing direct damage to the neurons, leading to an oligodendrocyte-independent axonal vulnerability. Although we cannot exclude the possibility of a direct cuprizone effect on neurons in our model, we hypothesize that due to the low ABCD1 expression [47] in neurons of the central nervous system, the difference observed between the genotypes is not primarily neuron-mediated.

Whether the oligodendrocyte pathology is a primary pathological process in X-ALD or occurs second to the immune response remains highly debated and may differ between CALD and adrenomyeloneuropathy. In the context of cuprizone-induced demyelination, there is evidence for microglia playing a direct role in driving oligodendrocyte death [48]. We observed similar dynamics of microglia recruitment and activation in *Abcd1* KO and WT mice during the demyelinating phase of the treatment. Although it is possible that the microglia are contributing to oligodendrocytic pathology in this model, we found no indication that the enhanced early loss of mature oligodendrocytes in *Abcd1* KO mice is driven by the microglia.

Efficient clearance of myelin from the lesions relies on the capacity of microglia to phagocytose and degrade the myelin debris [49]. *Post-mortem* analysis of CALD lesions have revealed the presence of particularly enlarged lipid-laden macrophages, suggesting a link between *ABCD1* deficiency and myelin clearance [15, 50]. Our present study revealed neither any major differences in the morphology of activated microglia between the genotypes nor in the extent of myelin loss. Thus, apparently, the ability of murine *Abcd1*-deficient microglia to clear out the myelin debris is not hindered by the genetic defect. The lack of genotype effect on microglial responses might be attributed to the structural and functional redundancy of peroxisomal fatty acid transporters (ABC transporter subfamily D). ABCD1 exhibits a high degree of overlap in substrate specificity with ABCD2, the closest homolog and one of three ABCD transporters found in peroxisomes [51–54]. Overexpression of ABCD2 has been shown to functionally compensate for ABCD1 function in human X-ALD fibroblasts in vitro [55] and in *Abcd1* KO mice [56]. In contrast to human X-ALD macrophages, murine macrophages express relatively high levels of *Abcd2* gene [14, 57]. Therefore, it is plausible that also in microglia the endogenous *Abcd2* expression is sufficient to compensate for *Abcd1* deficiency in the KO mice.

The role of astrocytes in the pathogenesis of X-ALD remains elusive. As expected in neurodegeneration, increased astrocyte activation is a feature also of X-ALD. In the context of CALD, astrocyte expression of stress-related heat shock proteins is elevated, even in pre-active CALD lesions [58]. An increased pro-inflammatory state has been described in various rodent and ABCD1-deficient astrocyte cell models in vitro [59, 60]. Metabolic and mitochondrial energy derangements causing oxidative stress in ABCD1-deficient astrocytes have been suggested to facilitate demyelination in human X-ALD [61]. However, in our mouse model, the activation of astrocytes in response to the cuprizone-induced demyelination progressed on schedule and to the same extent in both *Abcd1* KO and WT mice. This resembles our findings for microglia and in astrocytes high expression levels of *Abcd2* [62] may prevent severe functional deficits.

Understanding remyelination is crucial for devising novel therapeutic strategies based on its potential to protect axons from degeneration and reestablish proper nerve conduction [39]. In multiple sclerosis, denuded axons undergo successful remyelination across different disease stages [63, 64]. These remyelinated areas are subject to recurring episodes of demyelination and remyelination, resulting in remyelination failure and axonal degeneration, with the latter considered as the major correlate of clinical disability in MS [65]. Similarly, a recent study found that the extent of clinical disability in X-ALD patients correlates positively with the extent of axonal degeneration as measured by the released neurofilament light chain levels in the blood [66]. Whether remyelination is possible in X-ALD has been the issue of a long-standing debate. Bergner and colleagues showed the presence of myelinating, BCAS1^+^ oligodendrocytes in CALD lesions, but found no signs of successful remyelination [18]. In line with reports from the cuprizone literature, we observed a similar rise in OPC levels during the demyelinating phase in both genotypes, suggesting that *Abcd1* deficiency does not interfere with the recruitment and ability of OPCs to undergo proliferation in response to cuprizone intoxication. This observation can in part be accounted for by the apparently intact microglia function reflected in the similar microglial responses in *Abcd1* KO and WT mice. Microglia play a crucial role in promoting remyelination through myelin phagocytosis and the secretion of various factors which affect OPC function [67]. Myelin debris as such constitutes a major impediment to successful remyelination due to its inhibitory effect on OPC differentiation [68]. Therefore, interfering with the proper functioning of microglia/macrophages seriously compromises the remyelination process [49, 69].

Upon cuprizone withdrawal, we observed similar numbers of newly formed mature oligodendrocytes as well as a similar extent of remyelination in both genotypes. Therefore, we conclude that *Abcd1* deficiency does not affect the ability of OPCs to give rise to mature oligodendrocytes in this model. Considering the relatively high levels of *Abcd1* expressed in the OPCs during development, it remains unclear why their function would stay unperturbed by the genetic defect. It is possible that, due to the young adult age of the mice used in the cuprizone model, the metabolic effects of the genetic deficiency have not reached the levels sufficient to interfere with the functioning of the cell.

Concerning possible mechanisms that may accelerate oligodendrocyte cell death in *Abcd1* deficiency, ferroptosis is an interesting candidate. Ferroptosis, involving lipid peroxidation, iron metabolism and dysregulation of ferroptosis-counteracting enzymes, was recently reported as a contributor to pathology in X-ALD cells [70]. This mode of cell death is also featured in oligodendrocytes during week 2–4 of cuprizone intoxication [31] and, thus, well aligned with the time point showing enhanced loss of mature oligodendrocytes in *the Abcd1* KO mice.

Interestingly, we observed a lower degree of ongoing axonal damage in the corpus callosum of *Abcd1* KO compared to WT mice 5 days after cuprizone withdrawal. This observation was mirrored by lower levels of activated microglia in *Abcd1* KO mice compared to WT mice as well as a tendency towards less total (IBA1^+^) microglia staining within the lesions. One possible explanation for this could be that also the regenerative processes were initiated sooner in the KO mice, as a consequence of a more pronounced oligodendrocyte loss early in the demyelinating phase. Given the lack of genotype effect on microglia dynamics during the later stages of remyelination, this observation is more likely to be indicative of a subtle temporal shift in activation rather than genotype-specific differences in the microglia per se.

One of the major limitations of the cuprizone model is the variability in the demyelinating treatment responses and the overlapping onset of remyelination, possibly obscuring the less robust genotype differences that may still be biologically relevant. In our study, the biological variability affected both genotypes to a similar extent and could, at least in part, be the result of differences in cuprizone intake. Monitoring the exact cuprizone intake on an individual level, however, is difficult in group-housed mice. Furthermore, human and murine species differences in the expression levels of the peroxisomal transporters and in the immune system limit the interpretation of the cell type contributions for the human X-ALD.

Taken together, our data indicate that *Abcd1* KO mice are more susceptible to cuprizone-induced oligodendrocyte death and associated axonopathy. Future studies should unravel the molecular underpinnings of the responses that we observed in mature oligodendrocytes. In contrast to single *Abcd1* deficiency, an exacerbated metabolic phenotype was described in the CNS and isolated peritoneal macrophages of *Abcd1/Abcd2* double-knockout mice [57] and *Abcd1/Abcd2* double-knockout BV2 microglia cell line [71]. Given the lack of compensatory ABCD2 function in human phagocytes, understanding the dynamics between microglia and oligodendrocytes in cuprizone-treated mice under conditions of *Abcd1/Abcd2-*double deficiency may provide additional insights concerning microglia/macrophage-related aspects of CALD.

## Conclusion

Our study shows that *Abcd1* deficient mice respond more strongly to cuprizone-induced demyelination with a greater extent of both mature oligodendrocyte loss and acute axonal damage early in the demyelinating phase compared to wild-type mice. Previous observations from human, *post-mortem* CALD tissue suggested that oligodendrocyte-axonal disturbances precede the full-blown inflammatory reaction, in line with a primary genotype effect on oligodendrocyte function. Therefore, understanding the repercussions of the genetic *ABCD1* deficiency in X-ALD on oligodendrocyte physiology in the context of demyelination might provide the groundwork for novel therapeutic strategies.

## Supplementary Information

**Additional file 1: Table S1**. Primer sequences used in RT-qPCR.

**Additional file 2: Figure S1**. Old *Abcd1* KO mice show no signs of overt pathology in the corpus callosum. **Figure S2**. *Abcd1* KO mice exhibit the expected response to acute cuprizone intoxication. **Figure S3**. Gene expression analysis on dissected corpus callosum from cuprizone-treated WT and *Abcd1* KO mice.

## Data Availability

All data are presented in the manuscript and are available upon request.

## Abbreviations

ABCD1: ATP-binding cassette subfamily D member 1
*Aif1*: Allograft inflammatory factor 1, alias Iba1
APP: Amyloid precursor protein
CAII: Carbonic anhydrase II (protein)
CALD: Cerebral ALD
*Car2*: Carbonic anhydrase 2 (gene)
DAB: 3,3′-Diaminobenzidine
GFAP: Glial fibrillary acidic protein
H&E: Hematoxylin and eosin
*Hprt*: Hypoxanthine guanine phosphoribosyltransferase
IBA1: Ionized calcium-binding adaptor molecule 1
IHC: Immunohistochemistry
LFB/PAS: Luxol fast blue/Periodic acid Schiff
OLIG2: Oligodendrocyte transcription factor 2
OPC: Oligodendrocyte precursor cell
PFA: Paraformaldehyde
PLP: Proteolipid protein
VLCFAs: Very long-chain fatty acids
X-ALD: X-linked adrenoleukodystrophy
