# Supplementary material for: Abcd1 deficiency accelerates cuprizone-induced oligodendrocyte loss and axonopathy in a demyelinating mouse model of X-linked adrenoleukodystrophy
Source: Acta Neuropathol Commun. 2023 Jun 18;11:98. doi: 10.1186/s40478-023-01595-w (PMC10276915; doi:10.1186/s40478-023-01595-w)
Supplement: Supplementary file 1 — Additional file 1: Table S1. Primer sequences used in RT-qPCR. [file 40478_2023_1595_MOESM1_ESM.pdf]

Table S1. Primer sequences used in RT-qPCR.

Gene symbol	Forward primer	Reverse primer
Car2	5´-caagcacaacggaccaga-3´	5´-gtcctcctttcagcactgca-3´
Olig2	5´-atcttcctccagcacctcct-3´	5´-ccagtcgcttcatctcctcc-3´
Plp1	5´-agctgagttccaaatgaccttcc-3´	5´-gcgaagttgtaagtggcagc-3´
Aif1 (Iba1)	5´-agaagagactggggagctggt-3´	5´-ccaagtttctccagcattcgc-3´
Gfap	5´-gagcgtgcagagatgatgga-3´	5´-ggttggtttcatcttggagcttc-3´
Hprt	5´-acttcagggatttgaatcacgtt-3´	5´-gcagatggccacaggactaga-3´
