# Supplementary material for: Abcd1 deficiency accelerates cuprizone-induced oligodendrocyte loss and axonopathy in a demyelinating mouse model of X-linked adrenoleukodystrophy
Source: Acta Neuropathol Commun. 2023 Jun 18;11:98. doi: 10.1186/s40478-023-01595-w (PMC10276915; doi:10.1186/s40478-023-01595-w)
Supplement: Supplementary file 2 — Additional file 2: Figure S1. Old Abcd1 KO mice show no signs of overt pathology in the corpus callosum. Figure S2. Abcd1 KO mice exhibit the expected response to acute cuprizone intoxication. Figure S3. Gene expression analysis on dissected corpus callosum from cuprizone-treated WT and Abcd1 KO mice. [file 40478_2023_1595_MOESM2_ESM.pdf]

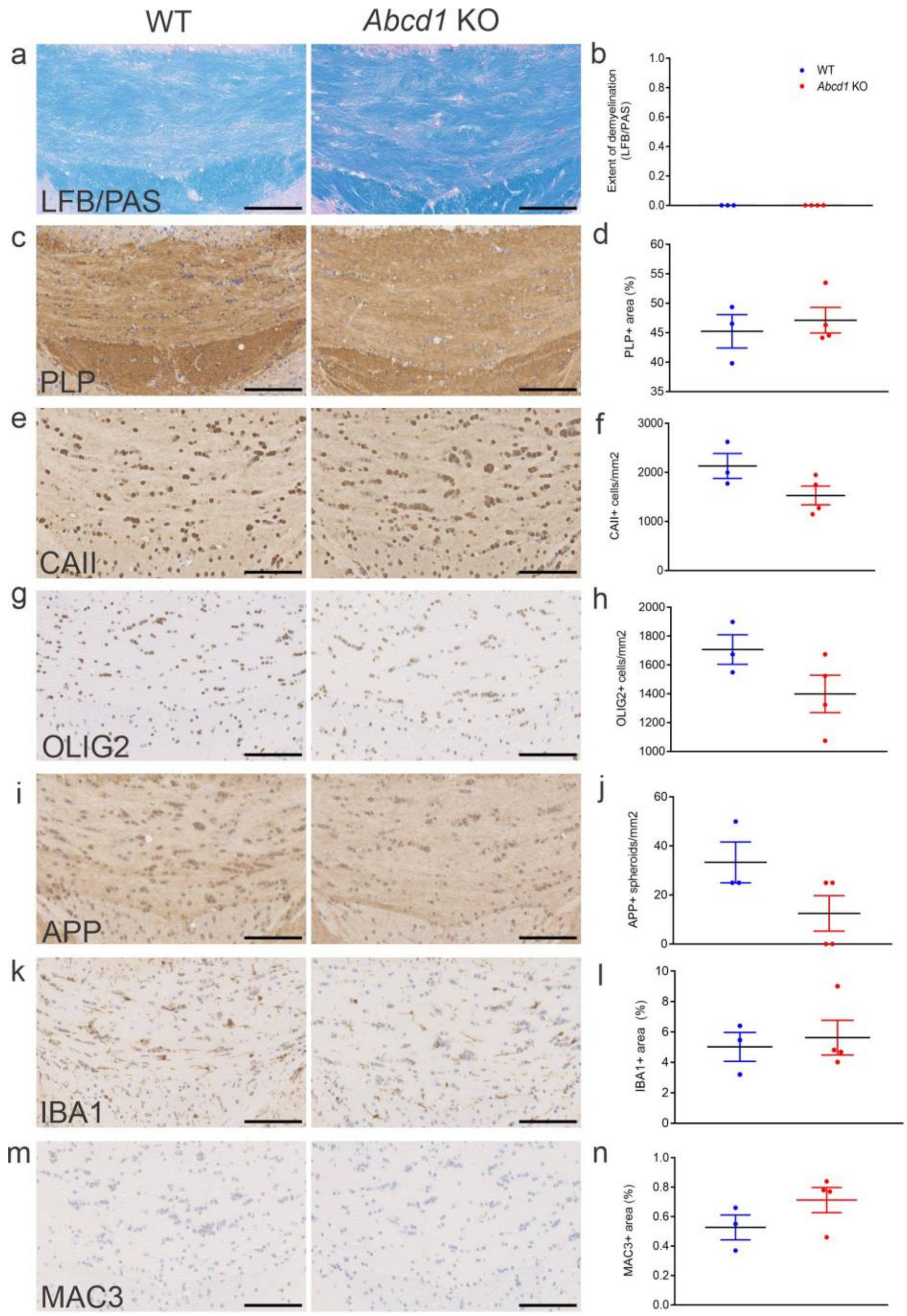

Fig S1. Old *Abcd1* KO mice show no signs of overt pathology in the corpus callosum. Representative images of the corpus callosum of 21-mon-old, untreated WT (*n*=3) and *Abcd1* KO (*n*=4) mice showing myelin stained with LFB/PAS or immunostaining for PLP (**a,c**), mature (CAII-positive) oligodendrocytes (**e**), all (OLIG2-positive) oligodendrocytes (**g**), acute axonal damage visualized as APP-positive spheroids (**i**), all (IBA1-positive) microglia (**k**) and activated (MAC3-positive) microglia (**m**) (scale bar: 100 µm). Graphs of the respective quantifications (**b, d, f, h, j, l, n**) show mean ± SEM.

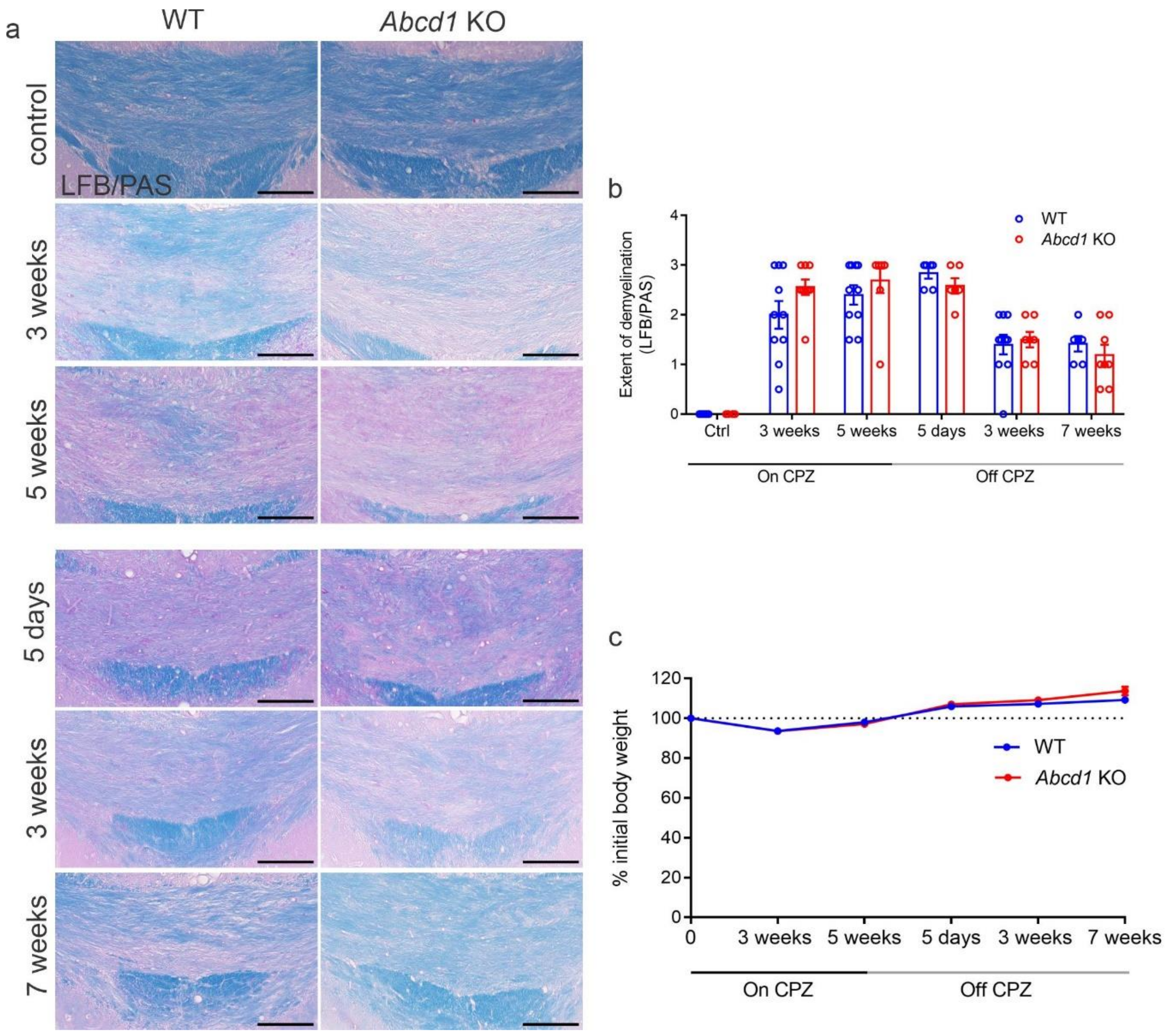

Fig S2. *Abcd1* KO mice exhibit the expected response to acute cuprizone intoxication. (**a**) Representative images of the LFB/PAS stained corpus callosum of WT and *Abcd1* KO mice across the experimental paradigm (untreated: *n*=5 WT, 4 *Abcd1* KO; treated: *n*=6-10 WT mice/time point and 6-9 *Abcd1* KO mice/time point). (**b**) Qualitative assessment of the extent of myelin loss and myelin renewal (intensity of LFB/PAS staining: 0 – no demyelination, 3 – complete demyelination) (**c**) Mouse body weight changes in response to 0.3% cuprizone feeding plotted relative to pretreatment weight (mean ± SEM).

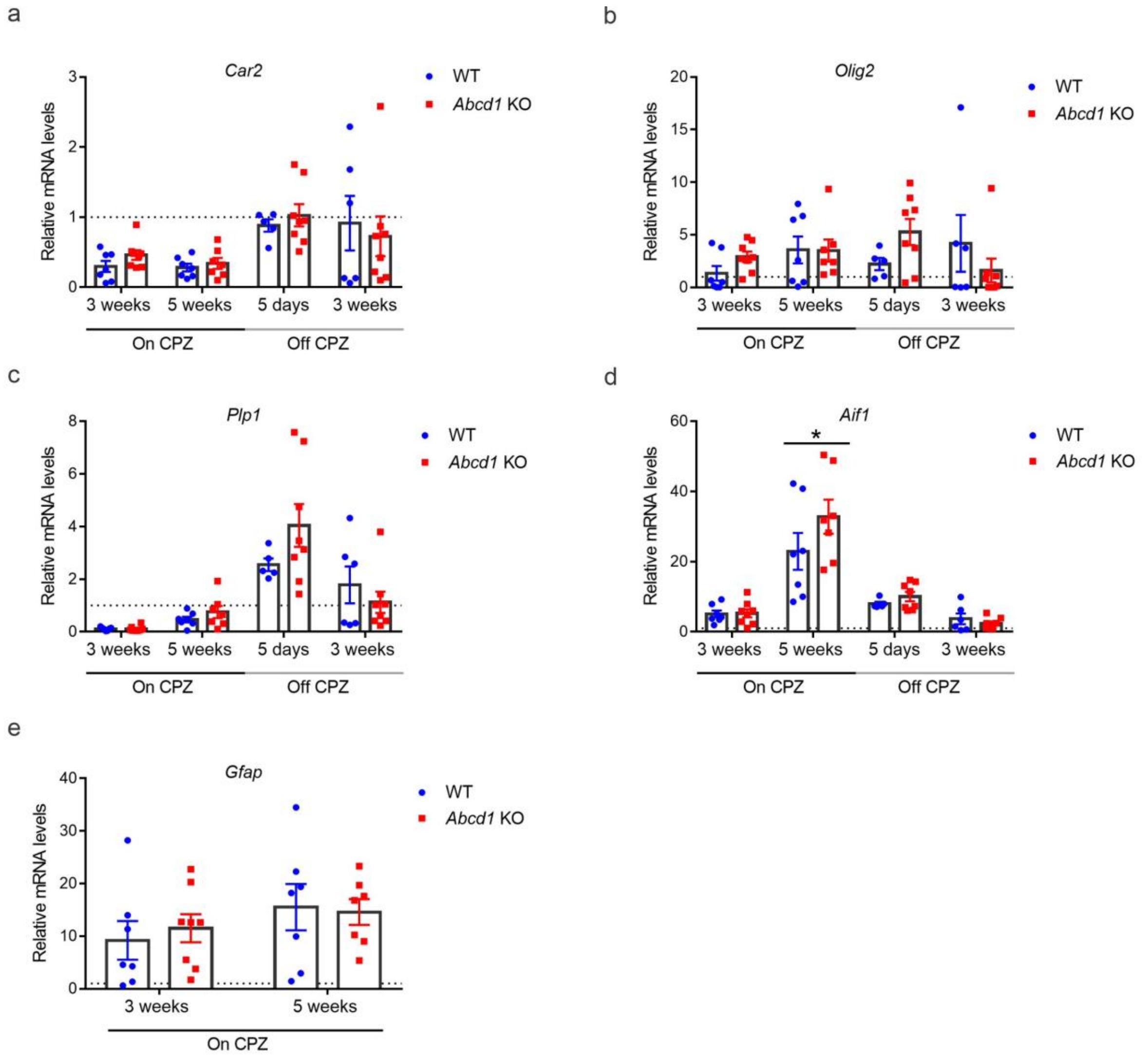

Fig S3. Gene expression analysis on dissected corpus callosum from cuprizone-treated WT and *Abcd1* KO mice. Analysis time points include 3 and 5 weeks on cuprizone, 5 days and 3 weeks after cuprizone withdrawal and untreated controls. Results of RT-qPCR for (**a**) *Car2,* (**b**) *Olig2,* (**c**) *Plp1,* (**d**) *Aif1/Iba1* and **(e)** *Gfap* are normalized to the mRNA levels of *Hprt* and calibrated to one untreated control (dotted line set to 1). Relative mRNA levels are shown as fold change to control. Statistical analysis: One-way ANOVA with Sidak's multiple comparisons test. Adjusted *p*-value: **p*=0.0497
